# 3D printing of mechanically functional meniscal tissue equivalents using high concentration extracellular matrix inks

**DOI:** 10.1016/j.mtbio.2023.100624

**Published:** 2023-04-05

**Authors:** Bin Wang, Xavier Barceló, Stanislas Von Euw, Daniel J. Kelly

**Affiliations:** a Trinity Centre for Biomedical Engineering, Trinity Biomedical Sciences Institute, Trinity College Dublin, Dublin, Ireland; b Department of Mechanical, Manufacturing and Biomedical Engineering, School of Engineering, Trinity College Dublin, Dublin, Ireland; c Department of Anatomy and Regenarative Medicine, Royal College of Surgeons in Ireland, Dublin, Ireland; d Advanced Materials and Bioengineering Research Centre (AMBER), SFI Research Centre for Advanced Materials and BioEngineering Research, Ireland

**Keywords:** Decellularization, ECM, Meniscus, Ink, 3D printing, Collagen alignment

## Abstract

Decellularized extracellular matrix (dECM) has emerged as a promising biomaterial in the fields of tissue engineering and regenerative medicine due to its ability to provide specific biochemical and biophysical cues supportive of the regeneration of diverse tissue types. Such biomaterials have also been used to produce tissue-specific inks and bioinks for 3D printing applications. However, a major limitation associated with the use of such dECM materials is their poor mechanical properties, which limits their use in load-bearing applications such as meniscus regeneration. In this study, native porcine menisci were solubilized and decellularized using different methods to produce highly concentrated dECM inks of differing biochemical content and printability. All dECM inks displayed shear thinning and thixotropic properties, with increased viscosity and improved printability observed at higher pH levels, enabling the 3D printing of anatomically defined meniscal implants. With additional crosslinking of the dECM inks following thermal gelation at pH 11, it was possible to fabricate highly elastic meniscal tissue equivalents with compressive mechanical properties similar to the native tissue. These improved mechanical properties at higher pH correlated with the development of a denser network of smaller diameter collagen fibers. These constructs also displayed repeatable loading and unloading curves when subjected to long-term cyclic compression tests. Moreover, the printing of dECM inks at the appropriate pH promoted a preferential alignment of the collagen fibers. Altogether, these findings demonstrate the potential of 3D printing of highly concentrated meniscus dECM inks to produce mechanically functional and biocompatible implants for meniscal tissue regeneration. This approach could be applied to a wide variety of different biological tissues, enabling the 3D printing of tissue mimics with diverse applications from tissue engineering to surgical planning.

## Graphical abstract

**Figure:**
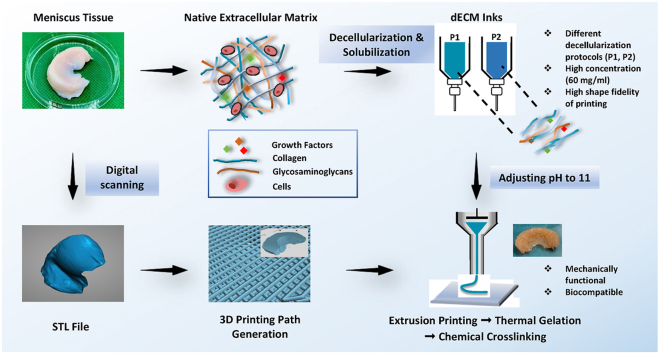

## 1 Introduction

Lesions to the meniscus are a common sports-related injury, which accounts for 15% of the injuries involving the knee joint [1]. The poor vascularization of this fibrocartilage tissue limits its healing capacity, and if left untreated can lead to cartilage degeneration and osteoarthritis [[2], [3], [4]]. Common surgical repairs provide a better clinical outcome compared to partial or total meniscectomies, however, there are still associated complications and risks with a large reoperation rate [2,5]. Allograft transplantation has been shown to delay the progression of joint degeneration and allows the majority of patients to gradually regain the ability to perform daily activities, but a shortage of donor menisci, challenges with the sizing of allograft meniscus and compatibility concerns makes this approach insufficient for the current and growing clinical requirements [[6], [7], [8]]. A number of meniscal scaffolds are in clinical use, notably the collagen meniscus implant [9] and the Actifit polyurethane meniscal scaffold [10], however these implants do not consistently promote meniscal regeneration [11]. Hence, there is therefore a distinct clinical need for new strategies for the repair of meniscal tissue, which has motivated the investigation of 3D printing technologies for the development of anatomically accurate, patient-specific implants that mimic the structure, composition and biomechanics of the native tissue.

In recent years, different 3D printing strategies have been developed that are capable of producing meniscal constructs recapitulating certain features of this fibrocartilaginous tissue [[12], [13], [14], [15], [16], [17]]. Synthetic polymers such as polycaprolactone (PCL) are commonly used to achieve the desired mechanical properties for *in vivo* applications [13,18,19]. For example, Lee and colleagues successfully 3D printed meniscus-shaped PCL scaffolds loaded with spatially defined microspheres containing transforming growth factor-β3 (TGF-β3) and connective tissue growth factor (CTGF), which supported hyaline cartilage-like tissue in the inner region of the implant and a fibrocartilage phenotype in the outer region [12]. Although the bioactive factors successfully induced the formation of meniscal tissue within the scaffold, the relatively high mechanical stiffness of the PCL implant resulted in degeneration of the underlying articular cartilage [20]. A number of studies have therefore explored varying different geometrical features like the polymer filament spacing and printed fiber diameter in an attempt to obtain scaffolds with more biomimetic mechanical properties [[21], [22], [23]]. However, the slow degradation rate of such biomaterials, a failure to produce constructs that are stiff in tension and soft in compression, and the relatively non-compliant nature of resulting implants motivates the development of alternative manufacturing methods and biomaterials for the development of more biomimetic meniscus implants [24].

Extracellular matrix (ECM) derived biomaterials have a well-established track record in the field of tissue engineering and regenerative medicine, and have recently been explored as potential inks for 3D (bio)printing [19,[25], [26], [27], [28], [29]]. The highly specialized ECM of the meniscus is critical to support its biological and biomechanical functions within the knee joint [30]. Therefore, 3D printing of anatomically accurate grafts that recapitulate normal meniscus composition, structure and biomechanics using tissue-specific decellularized ECM (dECM) inks represents a promising alternative to meniscal replacement implants currently in clinical use. Furthermore, meniscus-derived ECM materials have been shown to be more supportive of a meniscal phenotype compared to collagen type I scaffolds, further motivating the use of such ECM-derived biomaterials for meniscus repair applications [31]. While several meniscus ECM-derived materials have been reported, their use as a meniscus replacement remains challenging because of the difficulties in reproducing the anatomical structure of the native tissue [[32], [33], [34]]. One way to overcome this limitation is through 3D printing, which enables the patterning of different biomaterials into predetermined 3D architectures [35,36]. Moreover, a major limitation associated with dECM inks is their poor mechanical properties [37], making them unsuitable for immediate load-bearing applications, motivating research into new strategies to improve their mechanical properties.

**Fig. 1:**
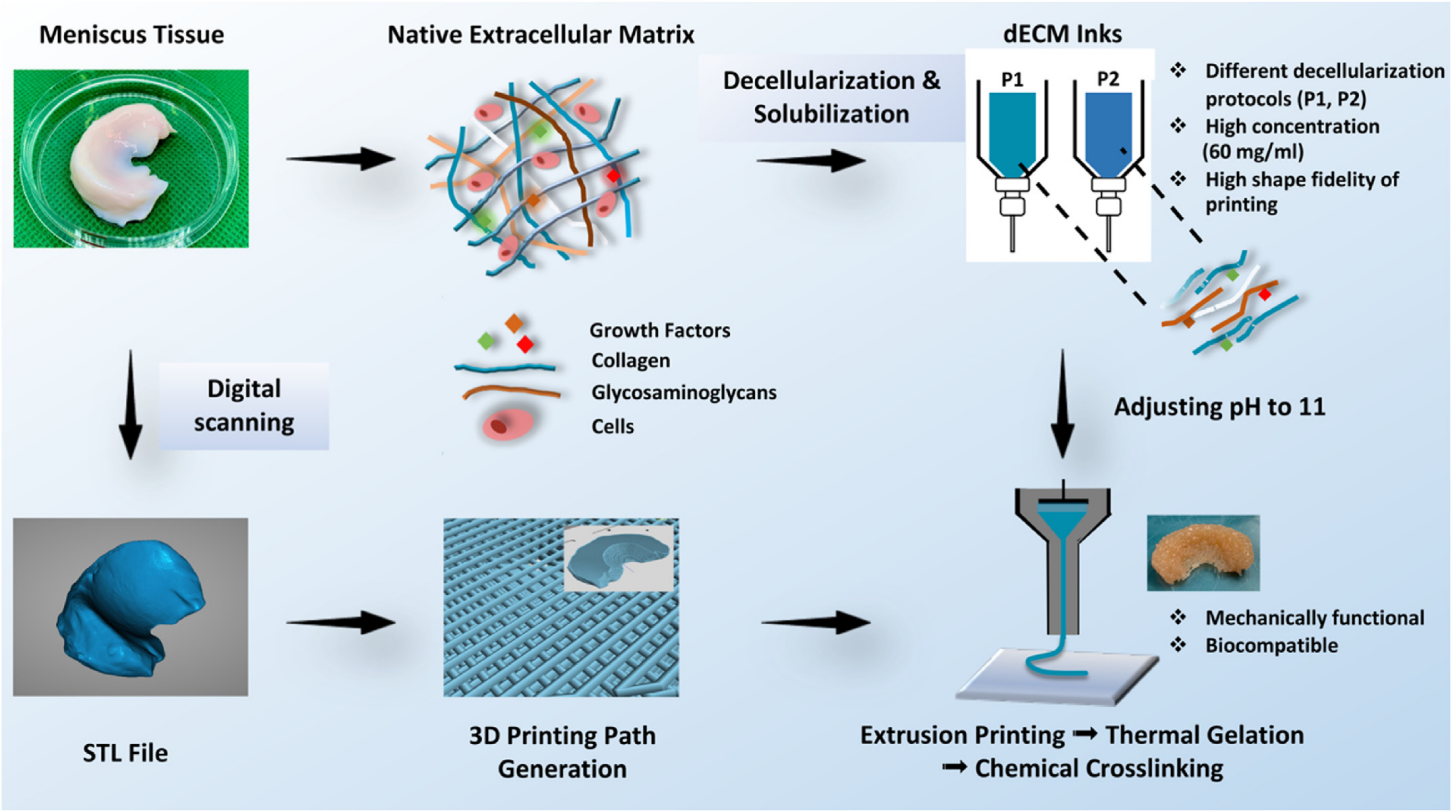
Schematic representation of mechanically functional meniscus implants printing using high concentration dECM ink. Native meniscus tissues were decellularized and solubilized with two different processes, high concentration dECM inks (60 ​mg/mL) were prepared in acidic condition. A porcine meniscus was scanned and converted into an STL file, and subsequently further generated printing path. Implants printing was performed with pH adjusted dECM inks and 3D path, followed by thermal gelation at 37 ​°C and glutaraldehyde crosslinking to obtain mechanically functional implants.

Here, we report the development of decellularized meniscus ECM-derived inks with rheological properties that impart excellent printability, enabling the fabrication of mechanically functional meniscal tissue equivalents (Fig. 1). Different decellularization protocols were investigated to access their effects on the biochemical composition of the final biomaterials. By modulating the pH of the dECM inks before printing and crosslinking, we demonstrate that it is possible to improve the printability of the dECM inks and produce mechanically robust and elastic ECM-derived constructs. The biocompatibility of the dECM constructs was further evaluated *in vitro* with porcine-derived fibrochondrocytes. Finally, we demonstrate that it is also possible to align the collagen fibers within the inks using extrusion-based printing at specific pH.

## 2 Materials and methods

### 2.1 Decellularization and solubilisation of meniscus tissue

Meniscus was harvested from 3 month old female pigs and diced into 1–2 ​mm pieces using a scalpel, followed by freeze-drying for 24 ​h at −10 ​°C and cryo-milled in liquid nitrogen. The powder of meniscus tissue was then decellularized with two different protocols. The tissue was first decellularized with protocol 1 (P1), which has been widely used for developing dECM inks [38,39]. Briefly, the powder was immersed in 10 ​mM Tris-HCl buffer (Sigma-Aldrich, Ireland) and went through 3 freeze-thaw (−80 to 37 ​°C) cycles with the buffer changed every cycle. The tissue was then treated with 1% triton-x100 solution for 24 ​h under rotation at room temperature. After washing with ultra-pure water, the tissue was treated with 10 ​mM Tris-HCl containing 0.15 ​M NaCl, 2 ​mM MgCl_2_ and 50 U/ml DNase (Sigma-Aldrich, Ireland) for 24 ​h at room temperature with gentle agitation, followed by 2 times of freeze-thaw cycles in 50 ​mM Tris-HCl buffer. The decellularized tissue was subsequently solubilized using a solution of 1500 U/ml of pepsin (Sigma-Aldrich, Ireland) in 0.5 ​M acetic acid under rotation for 24 ​h at room temperature. The tissue sample was centrifuged at 2500 ​g for 1 ​h to remove the insoluble material and the supernatant containing the solubilized tissue was transferred into a dialysis membrane (MWCO 12–14 ​kDa). The tissue sample was dialyzed against deionized water for 24 ​h before freeze-dried. The obtained dry decellularized tissue samples were kept at −85 ​°C for long-term usage.

The meniscus tissue was also decellularized with protocol 2 (P2), as previously described [40]. Briefly, the freeze-dried tissue powder was pretreated with 0.2 ​M NaOH solution for 24 ​h at 4 ​°C with gentle agitation to extract the majority of sulfated glycosaminoglycan (sGAG). After that, the tissue was subsequently solubilized with pepsin as described above, followed by a centrifugation step to remove insoluble material. The supernatant was combined with a 5 ​M NaCl solution to a final concentration of 0.8 ​M NaCl to preferentially salt precipitate collagen from the sample. The precipitated material was then solubilized again in 0.5 ​M acetic acid and the salt precipitation procedure was repeated a second time. The acid solubilized sample was then dialyzed against 0.02 ​M Na_2_HPO_4_ for 24h before lyophilized.

### 2.2 Histological and biochemical characterization of the dECM material

To verify the extent of decellularization, histological analyses and biochemical assays were performed to evaluate the content of DNA, sGAG and collagen in the decellularized tissue. For histological evaluation, both native and decellularized tissues were fixed in paraformaldehyde, embedded in paraffin wax, sectioned at 6 ​μm with a microtome (Leica, Germany). The sections were then stained with alcian blue to access sGAG content and picro-sirius red for collagen.

For biochemical analysis, the dECM samples and native tissue were enzymatically digested with papain solution (125 ​μg/mL papain in 0.1 ​M sodium phosphate with 5 ​mM Na_2_-EDTA and 5 ​mM cysteine-HCl at pH 6.5) for 16 ​h at 60 ​°C. DNA content was quantified using a Quant-iT Pico Green dsDNA assay kit (Invitrogen) according to the manufacturer's protocol. Quantification of sGAG in the digested samples were performed using a dimethylmethylene blue (DMMB) assay (Blyscan sulfated GAG assay kit, Biocolor). Collagen content was indirectly quantified by measuring hydroxyproline content using a Chloramine-T assay, assuming a hydroxyproline to collagen ratio of 1:7.69 [41].

### 2.3 dECM inks preparation

The dECM materials from P1 and P2 were dissolved in 0.5 ​M acetic acid at a concentration of 6% (w/v) at 4 ​°C for two days with rotation, the pH of the dECM inks was adjusted to 7.4 or 11 with 10 ​M NaOH using a dual syringe approach to obtain homogeneous solution before testing.

### 2.4 Rheological assessment of high concentration dECM inks

Rheological characterizations were conducted on an MCR 102 Rheometer (Anton-Paar, Hertford Herts, UK) equipped with a Peltier element for temperature control. A plate-plate geometry with a diameter of 25 ​mm (PP25) was used in all the tests. The viscosity as a function of shear rate (0.1–1000 s^−1^) was conducted at a constant temperature of 15 ​°C followed by sequential cycles of low (1 s^−1^) and high (100 s^−1^). Then, the gelation kinetics was assessed with a temperature sweep from 4 ​°C to 37 ​°C with an increment rate of 5 ​°C/min while maintaining the shear rate at 1 s^−1^. The frequency sweep (1–100 ​rad/s) was carried out after a 40 ​min of incubation at 37 ​°C at a constant strain of 2%. Bioinks were kept in a high humidity atmosphere to prevent dehydration from affecting the rheological results. All measurements were performed in triplicate.

### 2.5 3D printability of the high concentration dECM inks

The printability of the dECM inks with adjusted pH was accessed with the 3D Discovery printing system (Regen HU, Switzerland). The inks were loaded into a syringe and centrifuged to remove any air bubbles, and then printed at room temperature, using an extrusion pressure of 0.05–0.1 ​MPa, 10 ​mm/s translation speed and a 25 ​gauge needle, while the filament was deposited on a plate with temperature controlled at 37 ​°C. The printability of the inks was evaluated by measuring the spreading ratio, defined as the width of the filament divided by the diameter of the needle, as previously described [40]. In order to print a full size meniscus, a porcine meniscus was scanned and converted into an STL file, and subsequently further generated printing path with software. P1 pH11 inks was finally used to print the meniscus.

### 2.6 Mechanical characterization

Cylindrical constructs (5 ​mm diameter and 2 ​mm height) were fabricated by casting inks into custom designed PDMS molds, followed by thermal gelation at 37 ​°C for 4 ​h. After that, the cylinders were removed from the molds and immersed in 0.5% glutaraldehyde in ethanol to crosslink overnight at room temperature. The constructs were rinsed and equilibrated overnight with Dulbecco's Phosphate Buffer (PBS) before mechanical testing. All the mechanical characterization was performed at room temperature using a single column Zwick testing machine (Zwick, Roell, Herefordshire, UK) equipped with a 10 ​N load cell. Unconfined compression tests were carried out in a PBS bath. To ensure contact between the surface of the constructs and the top compression platen, a preload of 0.05 ​N was used. A peak of 10% strain was reached within 500 ​s and the equilibrium stress was obtained after a relaxation time of 20 ​min. After the relaxation phase, five compression cycles at 1% strain at a frequency of 1 ​Hz were superimposed. The ramp modulus was calculated as the slope of the initial linear region of the obtained stress-strain curves [42]. The equilibrium modulus was determined as the equilibrium force divided by the sample's cross-sectional area divided by the applied strain. The dynamic modulus was measured as the average force amplitude over the five cycles divided by the sample's cross-sectional area divided by the applied strain amplitude. Uniaxial tensile tests on casted samples were also performed. Tensile specimens were tested to failure at a displacement rate of 1 ​mm/min. The tensile modulus was taken as the slope of the stress-strain plots. To evaluate the elasticity of the constructs, samples were subjected to four compression cycles with strain amplitude of 10, 20, 30 and 40 in sequence. Construct permanent deformation (PD) after four cycles was calculated as follows: PD = (Test Speed ∗ Δt)/h_0_, where Δt is the interval of time at the start of the 4th cycle in which no force is applied to the sample, while h_0_ is the initial height of the sample [43].

### 2.7 Scanning helium ion microscopy (SHIM)

In order to observe the microstructure of the dECM constructs, the samples were fixed with 4% glutaraldehyde in PBS for 24 ​h at 4 ​°C. They were then washed with PBS, dehydrated by graded ethanol series and dried by critical point drying (EMS K850 Critical Point Dryer, Quorum Technologies Ltd., UK). SHIM was carried out on a ZEISS ORION NanoFab operating at 30 ​kV with a beam current of ≈2 ​pA. All the samples were coated with Gold/Palladium by sputtering an alloy target (Gold:Palladium, 60:40 ratio) prior to imaging.

### 2.8 Meniscal cell isolation

Medial and lateral knee menisci were obtained from a 4-month old pig. Under sterile conditions, the menisci were rinsed twice with PBS containing 100 U/mL penicillin and 100 ​μg/mL streptomycin (both Gibco, Biosciences, Dublin, Ireland). After 30 ​min of pronase digestion (500 U/mL) at 37 ​°C, inner and outer regions were plated separately and minced into pieces of 1–2 ​mm^3^. The tissue samples were then placed in a collagenase (Collagenase type I, Gibco) solution (900 U/mL) for 3 ​h at 37 ​°C under constant agitation. When samples were fully digested, the suspension was filtered through a 0.4 ​μm porous membrane, centrifuged and resuspended in standard culture media (XPAN) consisting of high glucose Dulbecco's modified Eagle's medium (hgDMEM) supplemented with 10% v/v FBS, 100 U/mL penicillin, 100 ​μg/mL streptomycin, and 2.5 ​μg/mL amphotericin B (all Gibco, Biosciences, Dublin, Ireland). Cells were then counted and seeded at the desired density. For the experiments, meniscus cells were used at passage 2.

### *2.9* *In vitro* analysis

The dECM constructs were sterilized with 70% ethanol, then rinsed and equilibrated in PBS overnight, firbrochondrocytes were seeded on top of the constructs at a density of 500,000 ​cells per sample. Constructs were cultured in XPAN medium. Cell viability was assessed at day 5 using live/dead staining (2 ​mM EthD-1 and 5 ​mM Calcein (Bioscience, UK) for 1 ​h) and confocal microscopy. To visualize cell attachment, cells were fixed with 4% paraformaldehyde PBS solution for 30 ​min, permeabilized in 0.5% Triton-X-100 solution for 10 ​min and then blocked in PBS containing 0.1% TX and 3% BSA at room temperature for 30 ​min, followed by incubating with 0.165 μM Alexa Fluor 660 Phalloidin (Thermo Fisher, Ireland) and 10 μg/mL 4′,6-diamidino-2-phenylindole (DAPI, Sigma-Aldrich, Ireland) for 1 ​h at room temperature. The cell morphology was observed through confocal microscopy. At each time point, the constructs were enzymatically digested with papain solution as described above, and the DNA content was accessed by a Quant-iT Pico Green dsDNA assay.

### 2.10 Alignment of collagen fiber through printing

P1 pH11 inks were printed through a 30 gauge needle, using an extrusion pressure of 0.5 ​MPa. The filament was deposited on a plate with temperature controlled at 37 ​°C. P1 pH 7.4 inks were also printed as a control. The filaments were fixed in paraformaldehyde, stained with picro-sirius red, and further observed with polarized light microscopy to investigate collagen fiber orientation. OrientationJ plugin from Image J was used to create the color maps of the fiber orientation, while directionality plugin was used to quantify the mean orientation and angular dispersion of the collagen fibers, as previously described [27].

### 2.11 Statistical analysis

All statistical analyses were performed using GraphPad Prism software. Numerical results were expressed as mean ​± ​standard deviation. Experiment groups were analyzed using either two-tailed Student's t-test or one-way analysis of variance (ANOVA) followed by Tukey's post-hoc test. Statistical significance was accepted at a level of p ​< ​0.05.

## 3 Results

### 3.1 Decellularization of native meniscus tissue

**Fig. 2:**
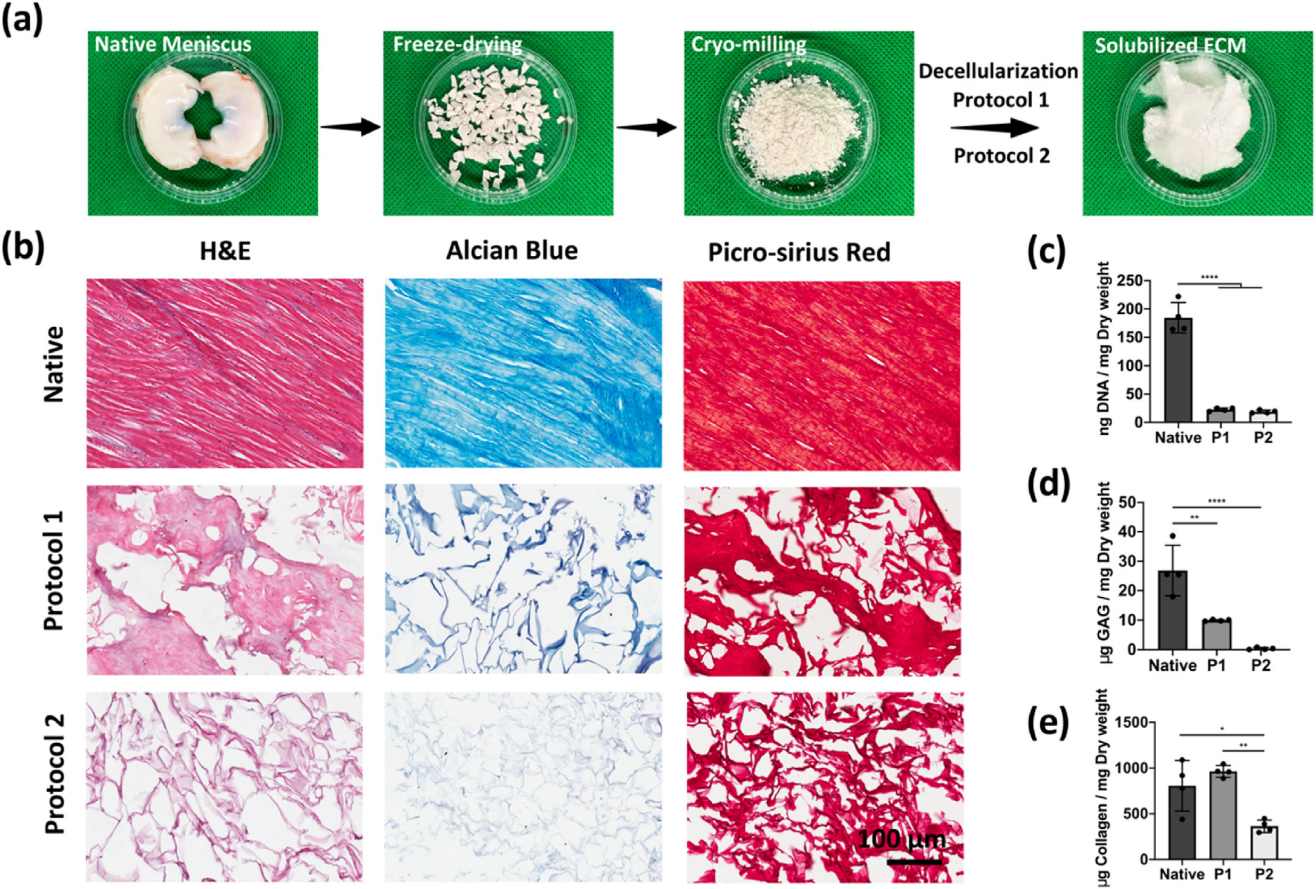
Decellularization of the native meniscus tissues and the histological and biochemical evaluations. (a) Macroscopic images of meniscus ECM during decellularization process. (b) Histological examination of dECM from two different protocols, stained with haematoxylin and eosin (H&E), alcian blue and picro-sirius red. Scale bar ​= ​100 ​μm. (c–e) Quantification of DNA, sGAG and collagen contents of native and decellularized tissues. n ​= ​4, ∗p ​< ​0.05, ∗∗p ​< ​0.01, and ∗∗∗∗p ​< ​0.0001.

The decellularization treatment is the first key step in the development of any dECM ink as it can affect the biochemical composition of the resulting material [40,44], which in turn affects the biological and mechanical functionality of the dECM-based constructs. For this reason, after the treatment process to obtain cryo-milled powder from the harvested tissue, the derived biomaterial was decellularized using two different protocols (Fig. 2a). Protocol 1 (P1) consisted of three cycles of freezing-thawing, followed by Triton-X-100, DNase, and pepsin solution treatment. On the other hand, protocol 2 (P2) consisted of NaOH treatment followed by pepsin digestion and salt precipitation. Even though both procedures yielded a similar level of decellularization as demonstrated by H&E staining and a significant reduction in DNA (Fig. 2b and c), the remaining sGAG and collagen content varies between the final products obtained from these two protocols. Histological analysis revealed a less intense alcian blue staining in P1 dECM compared to native tissue, while negligible alcian blue staining was observed in P2 products, which was further confirmed with a biochemical assay (Fig. 2d). In addition, significantly more collagen content was found in P1 products compared to that in P2 (Fig. 2e).

### 3.2 Rheological properties and 3D printability of dECM inks

**Fig. 3:**
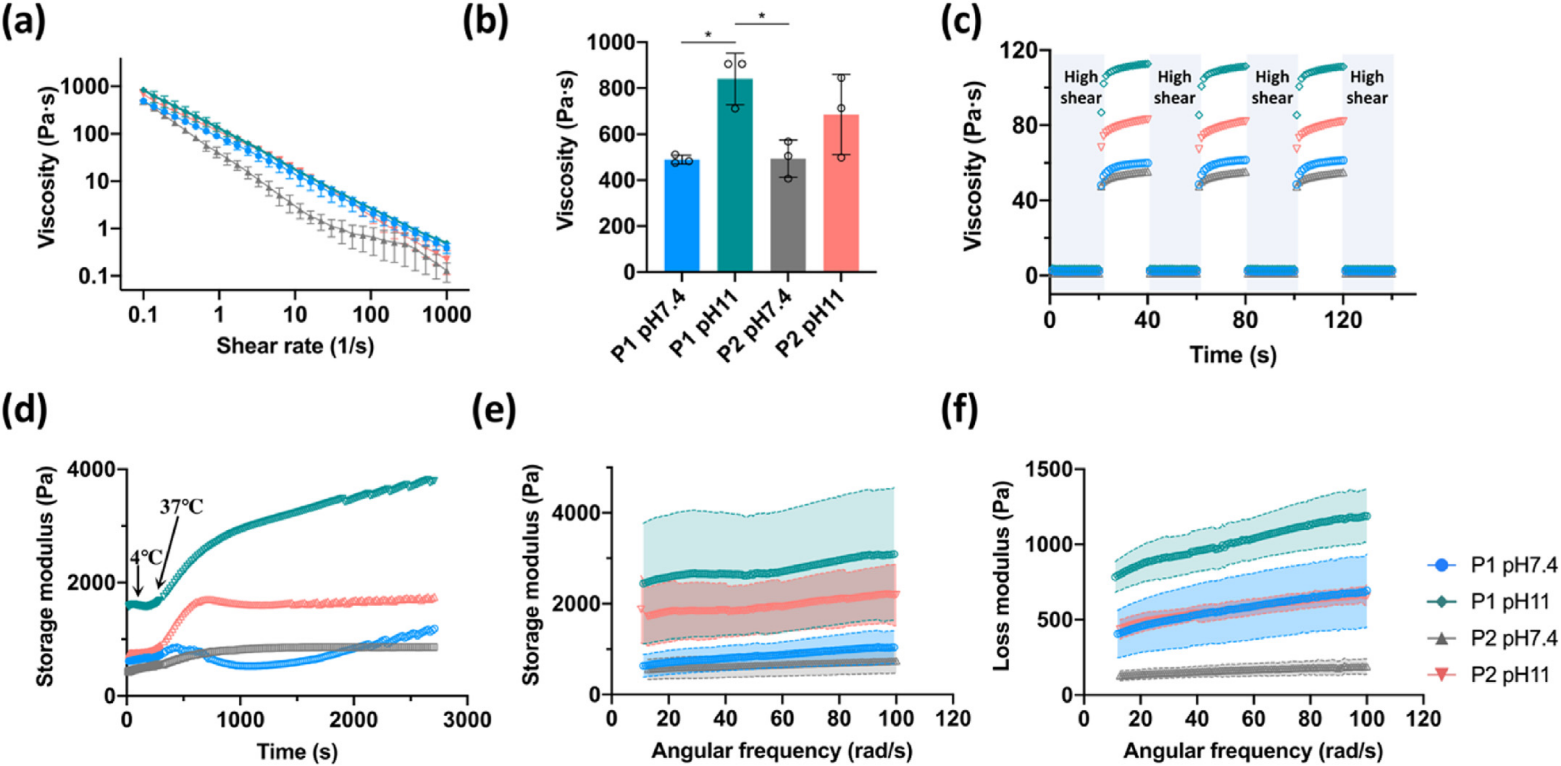
Rheological evaluation of dECM inks. (a) Viscosity of different bioinks in the presence of shear rate in the range of 0.1–1000 ​s^−1^ ​at 15 ​°C. (b) Viscosity of the dECM inks at the shear rate of 0.1 s^−1^. (c) Viscosity recovery test of the inks subjected to repeated cycles of low (1 ​s^−1^) and high shear rate (100 ​s^−1^). (d) Temperature sweep from 4 ​°C to 37 ​°C with increment of 5 ​°C min^−1^ and maintained at 37 ​°C for 40 ​min. (e, f) Storage and loss modulus at varying frequency at 37 ​°C. All experiments were performed in triplicate.

As pH is known to effect collagen fibrillogenesis *in vitro* [45], it seems reasonable to assume that pH levels will also influence the rheological properties and printability of dECM inks. The rheological characteristics of the high concentration of dECM inks at two different pH levels were next examined, including shear thinning behaviour, thixotropic property, and gelation kinetics. The shear-dependent viscosity of the different bioinks was acquired from shear rate sweep measurements (Fig. 3a). All dECM inks displayed shear-thinning behaviour at 15 ​°C, characterized by a continuous decrease in viscosity with increasing shear rate. However, the viscosity at a shear rate of 0.1 s^−1^ appeared to be significantly higher at pH 11 than that at pH 7.4 regardless of the decellularization protocol (Fig. 3b). The evaluation of thixotropic properties was also performed under alternating low/high shear rates (1 or 100 ​s^−1^) to simulate the printing process (Fig. 3c). All the different dECM inks went from fluid-like to solid-like behaviour in response to applied shear rates rapidly and repeatably. It was found again that the viscosity of the dECM inks at pH 11 was much higher compared to dECM inks with pH 7.4 when applied low shear rate. The kinetics of gelation was assessed through sets of temperature sweeps (Fig. 3d), consisting of an initial temperature ramp from 4 to 37 ​°C followed by an incubation for 40 ​min at 37 ​°C. At temperatures above 15 ​°C, the storage modulus of the dECM inks exhibited a pronounced increase, indicating the thermal gelation of the collagen-rich dECM inks with the increased temperature. After incubation at 37 ​°C, the storage modulus of P1 pH 11 and P2 pH 11 inks were found to be higher compared to dECM inks at a pH of 7.4. Moreover, the storage modulus of all the dECM inks was higher than the loss modulus, indicating the solid-like property of the dECM inks.

**Fig. 4:**
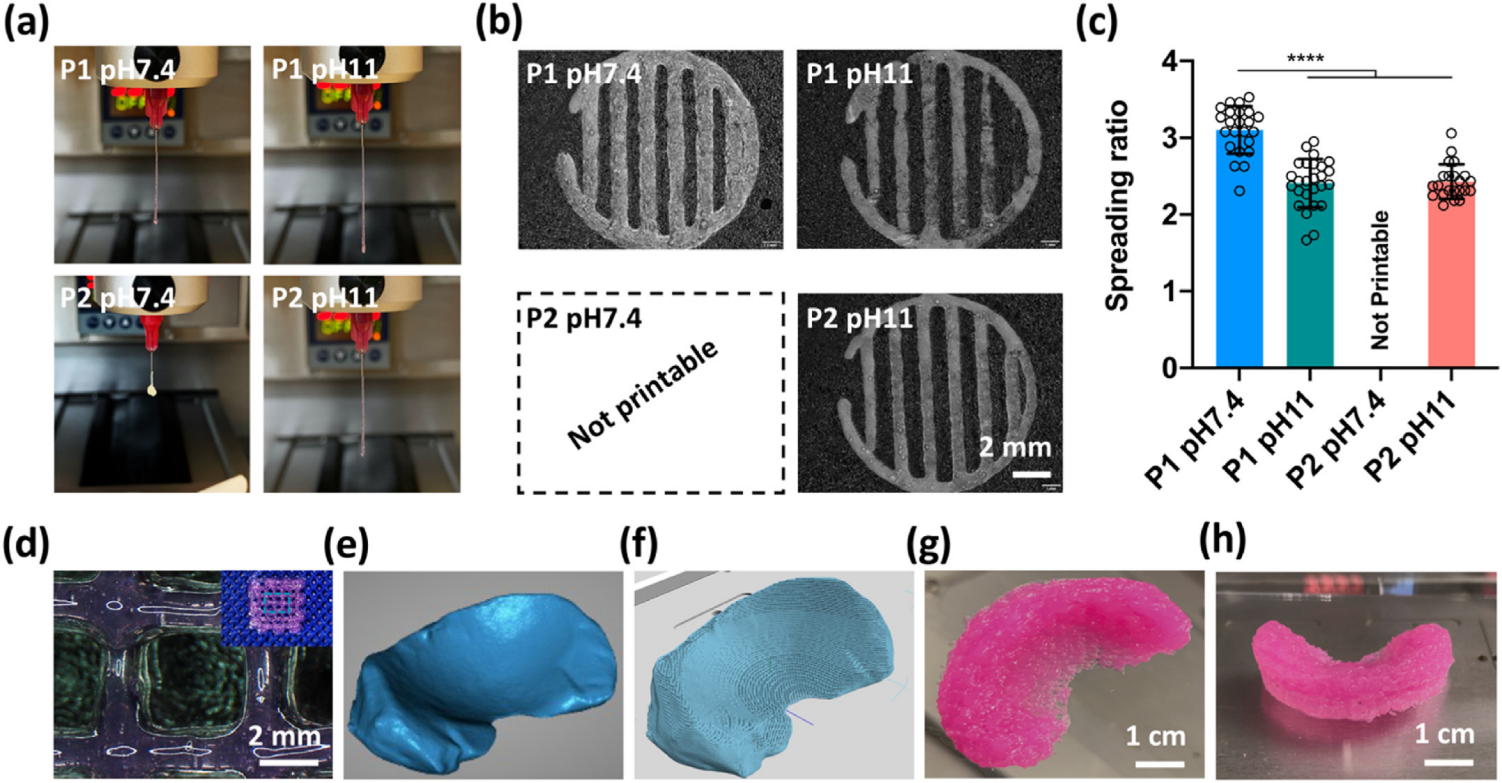
3D printability of dECM inks. (a) Macroscopic images of dECM inks extruded from a 25 gauge needle. (b) Printed pattern using different dECM inks. (C) Post printing spreading ratio (width of filament divided by needle diameter). ∗∗∗∗p ​< ​0.0001. (d) Printed grid structures with P1 pH 11 ink. (e) 3D model of a porcine meniscus. (f) Printing path for 3D printing. (g, h) Images of printed meniscus structures using P1 pH 11 ink.

The printability and shape fidelity of the dECM inks were further evaluated by characterization of the filament spreading ratio after deposition. For the four different derived inks, only the dECM ink obtained using P2 and at neutral pH could not be consistently extruded through a 25 gauge needle (Fig. 4a). On the contrary, all other inks could be easily extruded and patterned (Fig. 4b). The spreading ratio of dECM inks at pH 11 were consistently lower than that of those at neutral pH (pH 7.4), thus obtaining more smooth and uniform filaments (Fig. 4c). Grid-like structures were further printed using the P1 pH 11 ink, demonstrating again high print fidelity (Fig. 4d). After showing the ability to print simple structures, we looked into printing more clinically relevant sized constructs. A meniscus 3D model was generated from a porcine meniscus (Fig. 4e). This solid model was then sliced into multiple layers with the desired filling pattern and printed within 25 ​min, without collapse of the structure or clogging of the nozzle (Fig. 4f–h and Video S1).

Supplementary data related to this article can be found at https://doi.org/10.1016/j.mtbio.2023.100624.

The following is the supplementary data related to this article:

Video S1 Video S1

### 3.3 Mechanical properties, microstructure and biocompatibility of the dECM constructs

**Fig. 5:**
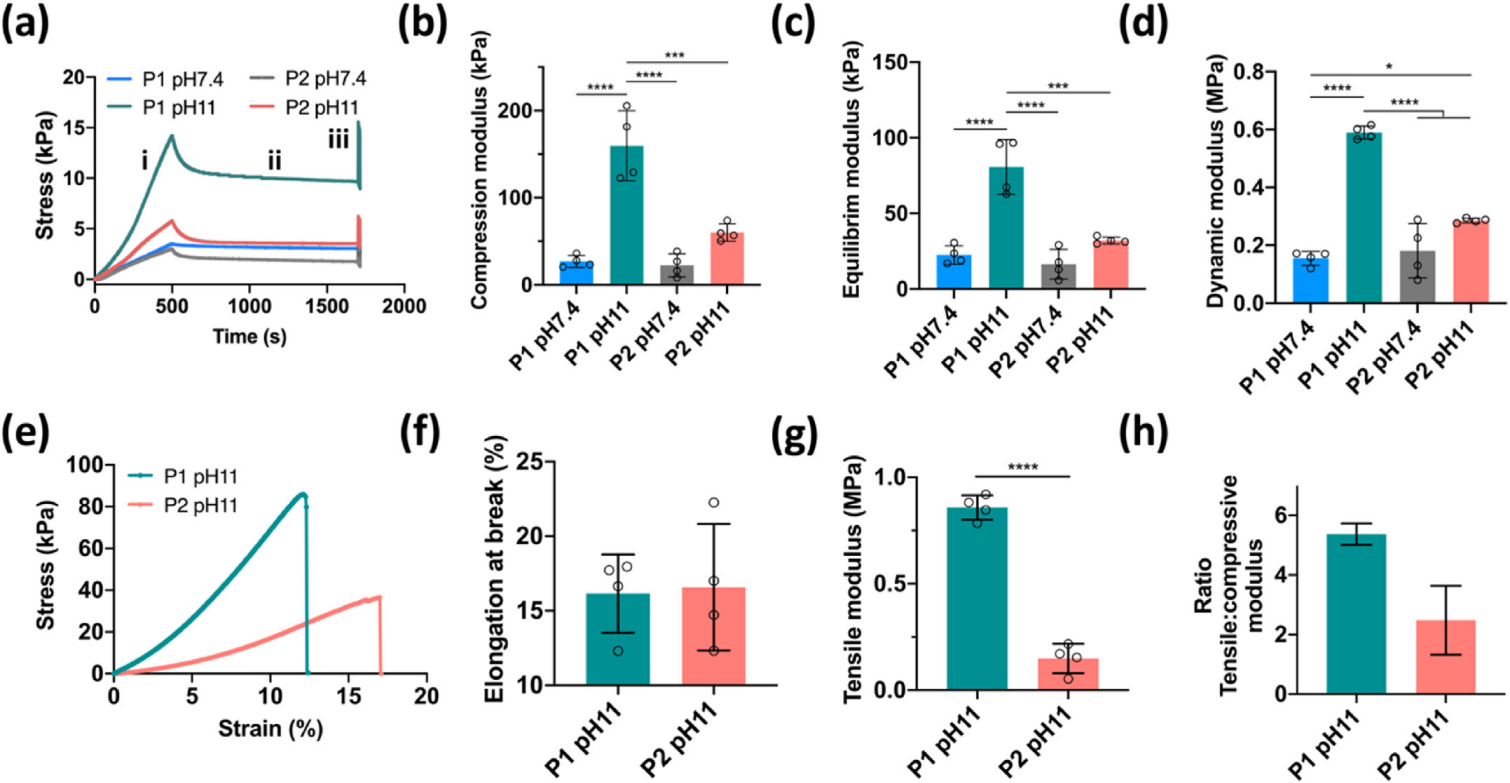
Mechanical properties of dECM constructs. (a) Representative stress-time curves of unconfined compression test consisted of (i) ramp compression to 10% strain, (ii) relaxation while keep 10% strain and (iii) cyclic loading at 1% amplitude and 1 ​Hz frequency. (b–d) Compression modulus, equilibrium modulus and dynamic modulus of the dECM constructs. (e) Representative stress-strain curves from tensile test. (f) Elongation at break and (g) tensile modulus of dECM constructs. (h) Ratio of tensile and compression modulus. n ​= ​4, ∗p ​< ​0.05, ∗∗∗p ​< ​0.001, and ∗∗∗∗p ​< ​0.0001.

Constructs generated using the dECM inks were cross-linked by immersion in 0.5% glutaraldehyde overnight at room temperature. In order to characterize the mechanical properties of the cross-linked dECM constructs, a stress-relaxation test followed by dynamic tests was first performed in an unconfined compression configuration (Fig. 5a). P1 pH 7.4 and P2 pH 7.4 appeared to have comparable compression modulus, whereas a significant increase was found when the pH of the dECM was adjusted to 11, regardless of the decellularization protocols. Additionally, P1 pH 11 constructs were found to have an over 2-fold higher compression modulus than P2 pH 11 (Fig. 5b). Similar difference were observed for the equilibrium and dynamic modulus (Fig. 5c–d). Having demonstrated superior mechanical properties at pH 11, tensile tests were performed on P1 pH 11 and P2 pH 11 constructs to assess their tensile properties and tension-compression nonlinearity. The results showed that both samples displayed comparable elongation at break (around 16%), while the tensile modulus of P1 pH 11 was 4-fold higher than P2 pH 11. When quantifying the ratio between tensile and compressive modulus, higher tension-compression nonlinearity was observed in P1 pH 11 constructs in comparison to P2 pH 11.

**Fig. 6:**
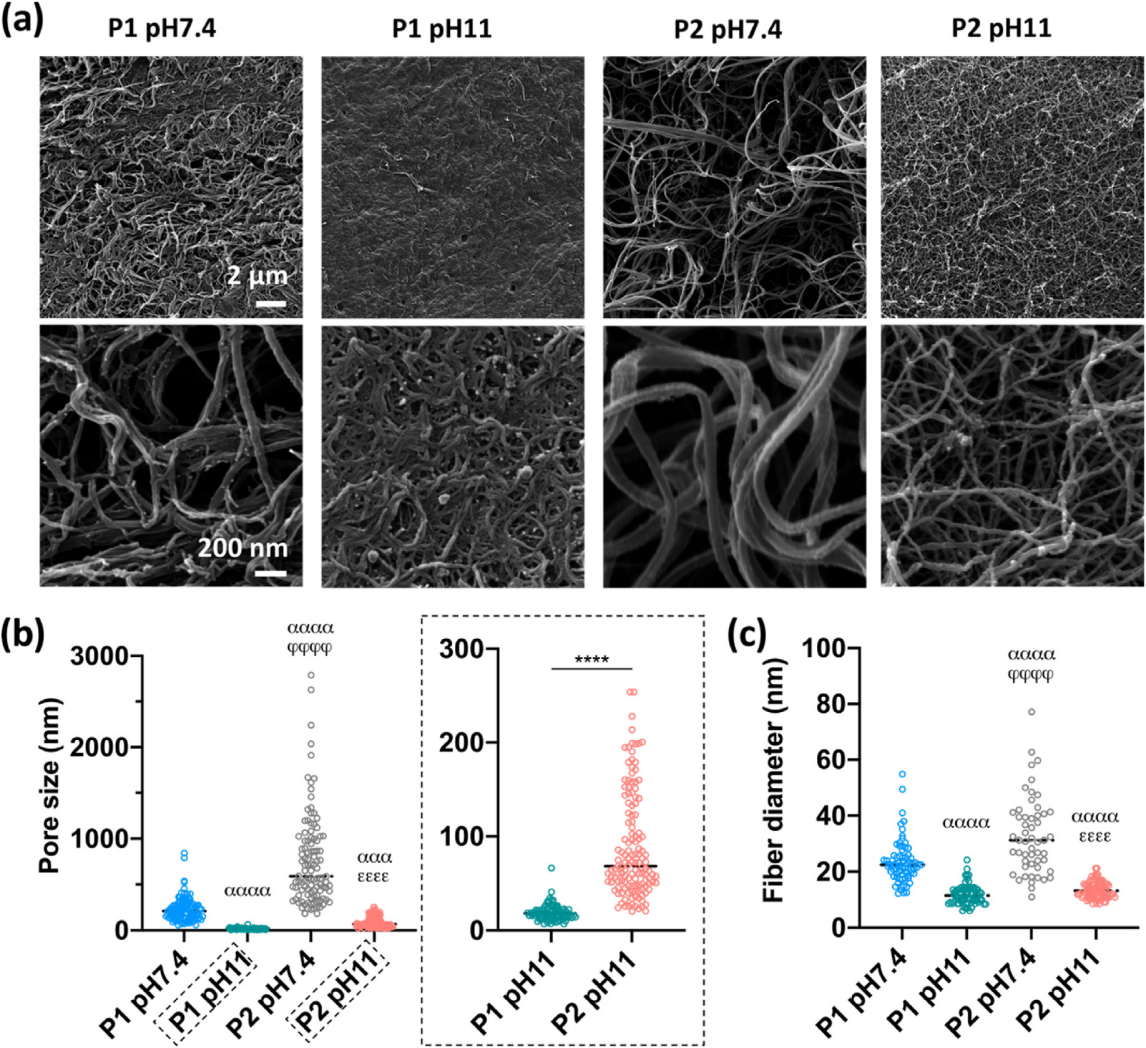
Microstructure of dECM constructs. (a) Scanning helium ion microscopic images of dECM constructs. (b) Pore size of the dECM constructs. (c) ECM fiber diameter of the constructs. ^***αααα***^p < 0.0001 vs. P1 pH 7.4, ^***φφφφ***^p < 0.0001 vs. P1 pH 11, ^***εεεε***^p ​< ​0.0001 vs. P2 pH 7.4, and ∗∗∗∗p ​< ​0.0001.

To further understand the differences in mechanical properties between groups, the microstructure of the different dECM constructs was assessed using scanning helium ion microscopy (Fig. 6a). The dECM constructs at pH 11 appeared to form more intense networks compared to those constructs at pH 7.4, regardless of the decellularization protocols. Moreover, a smaller pore size was found in P1 pH 11 constructs, possibly due to the relatively higher content of collagen in the dECM products derived from P1. These observations were further confirmed by the quantification of the pore size (Fig. 6b). Interestingly, collagen fibers were significantly smaller in diameter in constructs at pH 11 compared to those at pH 7.4 (Fig. 6c).

**Fig. 7:**
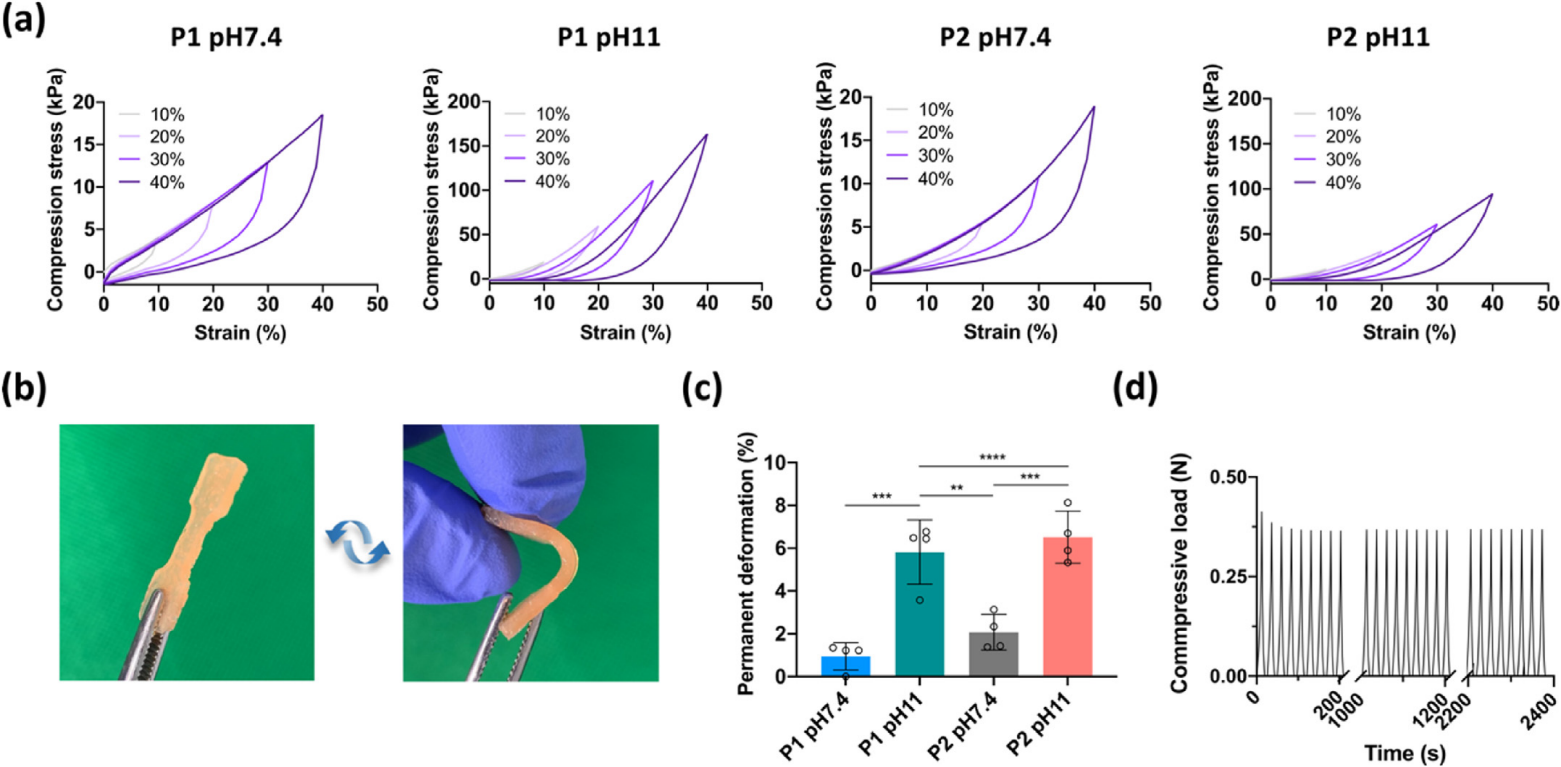
Elasticity evaluation of dECM constructs. (a) Representative stress-time curves of cyclic compression. (b) Macroscopic images of bending and recovery of P1 pH 11 dumbbell-shaped sample. (c) Permanent deformation of constructs after cyclic compression. n ​= ​4, ∗∗p ​< ​0.01, ∗∗∗p ​< ​0.001, and ∗∗∗∗p ​< ​0.0001. (d) Load profile of cyclic compression (100 cycles) at 10% strain.

The elasticity of the dECM constructs was evaluated by cyclic compression tests, which consisted of four cycles with increasing strain amplitude from 10% to 40%. All the constructs were shown to be resilient under cyclic loading, particularly the dECM constructs at pH 7.4, which exhibited negligible hysteresis and were able to recover to their original shape with only slight permanent deformations (less than 2%) (Fig. 7a and c). The elasticity of P1 pH11 samples was further demonstrated by a simple bending test, where the sample fully recovered after stress releasing (Fig. 7b and Video S2). To further investigate the long-term resilience and fatigue of P1 pH 11 constructs, long-term cyclic compression tests at 10% strain were performed to mimic the conditions at the site of implantation. The loading and unloading curves were almost identical, further demonstrating the highly elastic nature and stability of this dECM construct (Fig. 7d).

Supplementary data related to this article can be found at https://doi.org/10.1016/j.mtbio.2023.100624.

The following is the supplementary data related to this article:

Video S2 Video S2

**Fig. 8:**
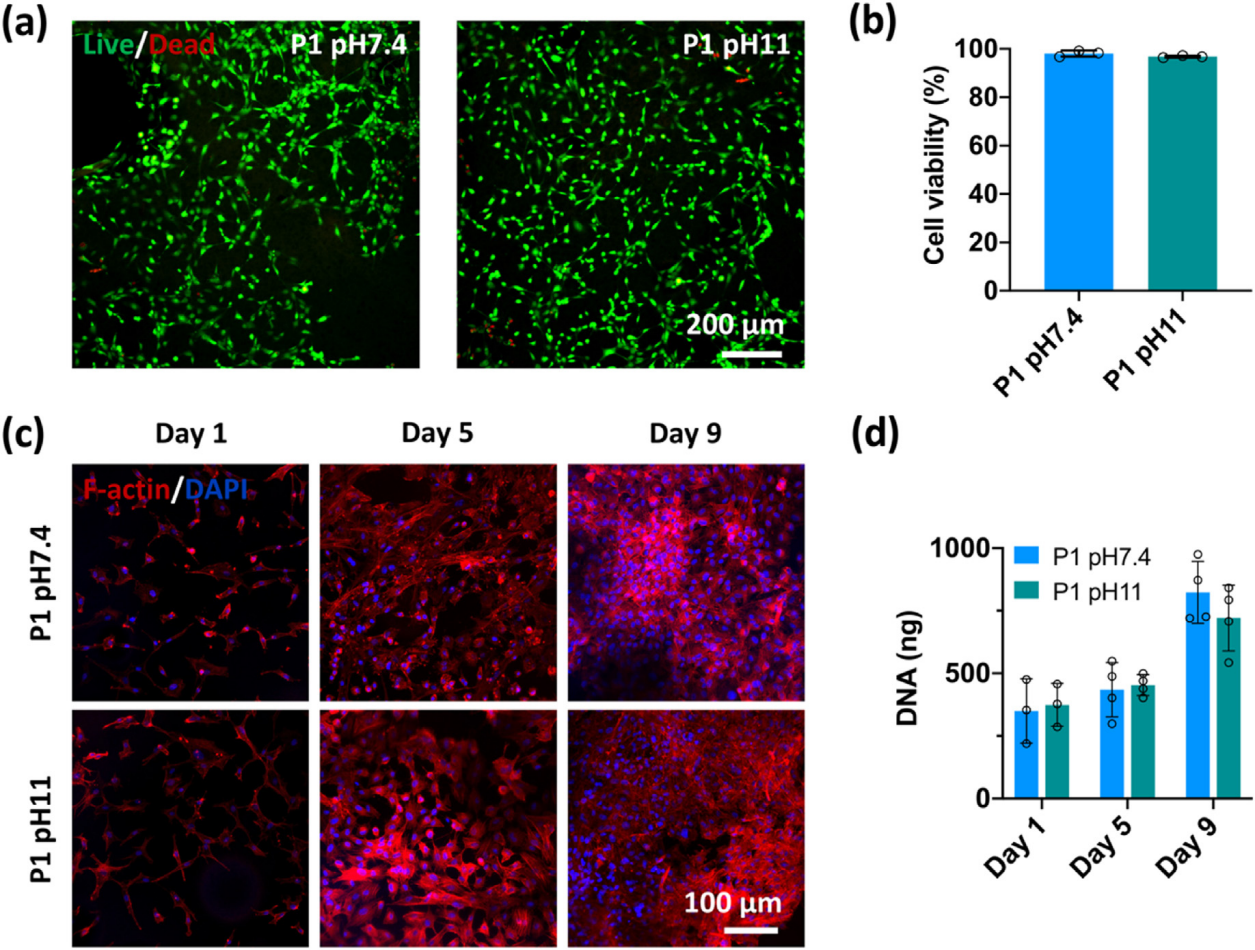
Biocompatibility evaluation of dECM constructs. (a) Representative images of live/dead staining of meniscus cells after seeded 5 days on dECM constructs. (b) Quantification of cell viability in 3D-printed constructs. 3 samples for each group analyzed. (c) Representative images of F-actin and DAPI staining of meniscus cells at various time points. (d) DNA contents quantification. n ​= ​3 or 4.

Having demonstrated the superior mechanical properties of the P1 pH 11 based biomaterials, the biocompatibility of this dECM construct was assessed by seeding them with primary porcine fibrochondrocytes, with P1 pH 7.4 constructs also evaluated as a control to assess the effect of pH on cell behaviour. A live/dead assay was performed on the dECM constructs after 5 days of seeding, which demonstrated limited cell death in both groups, with the cell viability of ∼95% (Fig. 8a and b). From the F-actin staining of the cells at various time points, it was observed that the cells were able to attach, spread and proliferate on both groups, which was further confirmed by a DNA assay which demonstrated an increase in cell number with time in culture. No significant difference in DNA content was found between the two groups, indicating that pH had little effect on the biocompatibility of the resulting dECM constructs.

### 3.4 Promoting alignment of collagen fibers within dECM inks using extrusion printing

**Fig. 9:**
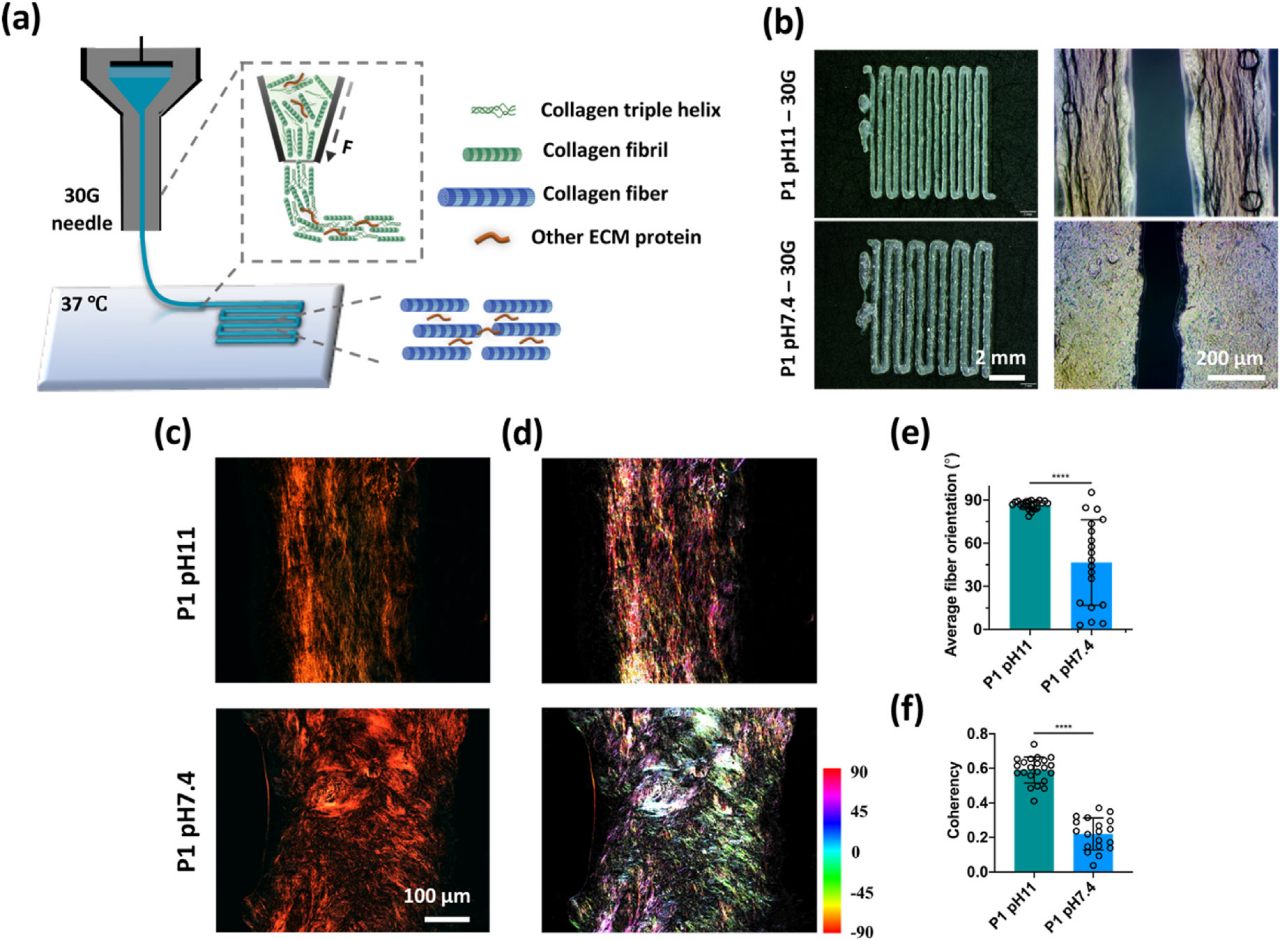
Alignment of collagen fiber through printing. (a) Schematics of 3D printing dECM inks for aligning collagen fibers. (b) Optical images of printed filaments using dECM inks with different pH. (c) Polarized light and (d) color map imaging of collagen fiber orientations in printed ECM filaments. (e, f) Quantifications of collagen fiber orientations and coherency. n ​= ​3 with multiple regions analyzed per sample, ∗∗∗∗p ​< ​0.0001.

With a view to mimicking the aligned collagen structure in the native meniscus tissue, we also examined if alignment of the collagen fibers within the dECM inks could be introduced through extrusion-based printing. In this case, a small size needle (30 gauge) was used to promote the alignment of the collagen fibrils, and the ECM was deposited on a plate with a temperature controller set at 37 ​°C to induce thermal gelation. The dECM inks from P1 at both pH could be printed and displayed high shape fidelity, however distinct differences in microstructure were observed, with the collagen network P1 pH 11 constructs appearing to be more aligned (Fig. 9b). This aligned structure was further confirmed with polarized light microscopy which allowed us to observe the organization of collagen fibers. The collagen fibers of the P1 pH 11 ink were preferentially oriented to the printing direction, with the fiber orientation close to 90°, whereas the collagen fibers within the ink at pH 7.4 appeared rather dispersed (Fig. 9c–e). Moreover, the coherency, a coefficient used to evaluate the variance of the fiber distribution, was significantly higher in P1 pH 11 constructs.

## 4 Discussion

Despite the tremendous potential of tissue engineering approaches for regenerating meniscal tissue, realizing an efficacious therapeutic strategy clinically remains elusive. The reasons for this are multifaceted, from the requirement for long *in vitro* maturation protocols for engineered tissues to poor neo-tissue formation after implantation of such grafts [46,47]. Therefore only relatively simple ‘off-the-shelf’ implants for meniscus repair, notably the collagen meniscus implant and the Actifit polyurethane meniscal scaffold [9,10], have seen clinical use. While these approaches hold great potential in the field of regenerative medicine, they are limited by the use of materials with relatively poor bioactivity, difficulties engineering anatomically accurate grafts, and the relatively poor mechanical properties of the implants compared to the native tissue which may limit the widespread clinical adoption of such techniques [48]. This study aimed to provide mechanically functional implant for meniscus repair by 3D printing a highly concentrated meniscus dECM ink. Native meniscus tissue was first decellularized and solubilized following two different protocols, which resulted in differences in the biochemical content, rheological properties and printability of the dECM inks. Modulation of the pH of the highly concentrated dECM inks was found to improve printability and the mechanical properties of the printed implants. Further crosslinking the dECM inks post-printing stabilized and enhanced the mechanical properties of the resulting constructs, while maintaining good biocompatibility. Finally, it was possible to align the collagen fibers by adjusting the pH of the dECM inks prior to extrusion printing, expanding the potential of the current strategy to print implants which also recapitulate the anisotropic microstructure of native meniscus tissue.

Implants made of tissue-specific dECM have been shown to recapitulate the inherent microenvironmental niche of cells, promoting a tissue-specific phenotype when seeded with stem/progenitor cells [27,49]. However, the composition of the final dECM products varies depending on the decellularization method [44,50], which in turn affects the rheological properties and printability of the developed dECM inks and the mechanical functionality of the printed grafts. In this study, the dECM from both protocols produced sufficiently decellularized products, with less than 50 ​ng DNA per mg of dry weight remaining in the biomaterials (a threshold that has been suggested for clinical applications of dECM scaffolds [51]). Nevertheless, significant differences in terms of sGAG and collagen content were found between the dECMs from these two protocols. Negligible levels of sGAG was found in the P2 dECM product compared to the P1 dECM product or the native tissue, possibly due to the treatment with NaOH which has been demonstrated to solubilise proteoglycans [51]. While it is well established that sGAG are integral to the compressive behaviour of the tissue [52], and this loss in sGAG may contribute to the lower mechanical properties of the P2 crosslinked constructs, the influence of such changes on the regenerative properties of these constructs is unclear. Therefore future *in vivo* work is required to compare the potential of dECM biomaterials generated from these two protocols to regenerate meniscus tissue. In addition, the collagen content in the dECM from these two protocols was significantly different, with around 3-fold more found in P1 dECM, which could be attributed to the relatively complex procedure involved in P2 leading to the loss of collagen. Another possible reason is the presence of residual salts in the final P2 products, which are required to reduce the precipitation of ECM [53].

Having demonstrated the successful decellularization of the native tissue, we next sought to investigate the rheological properties and printability of the high concentration dECM inks with adjusted pH. While dECM inks have gained broad interest in the field of tissue engineering, most published studies have focused on 3D bioprinting with cells, and in order to reduce viscosity and improve cell viability, relatively low ECM concentrations have been used [54,55]. With a view to 3D printing cell-free implants with mechanical properties compatible with implantation into load bearing environments, we instead explored the use of highly concentrated dECM inks which could also be chemically cross-linked post-posting. Previous studies have demonstrated that pH can affect the electrostatic interactions between collagen molecules and further influence the process of collagen fibrillogenesis [45,56]. Here, we found that at a low shear rate, the viscosity of the dECM inks at pH 11 was significantly higher than that of dECM inks at pH 7.4 (Fig. 3b–c), which may be correlated to altered fibrillogenesis of collagen at pH 11. Further, the increase of viscosity was associated with an improvement of the printability of dECM inks at pH 11, as evident by the decreased spreading ratio of the deposited filaments. It was also possible to print geometrically complex and large constructs, such as a full-size meniscus, using the high concentration of dECM inks at pH 11, which demonstrated the potential of the current strategy to print patient-specific implants. The investigation of gelation kinetics of the dECM inks also revealed that the storage modulus of dECM inks at pH 11 was higher than that of dECM inks at pH 7.4 even at a low temperature (4 ​°C), implying again the promotion of collagen fibrillogenesis at pH 11. Moreover, after thermal gelation, all the dECM inks, especially the dECM inks at pH 11, exhibited greater storage modulus than loss modulus, indicating the elastic nature of the resultant hydrogel network [56].

It is well known that the menisci are under constant mechanical stress and play a key role in joint biomechanics [11]. In order to enhance the mechanical properties of the dECM constructs, glutaraldehyde cross-linking was employed after thermal gelation as this has previously been employed to robustly crosslink collagen-based biomaterials by forming bonds between collagen molecules [57]. Significant differences in the compressive modulus was found between constructs prepared at pH 7.4 and pH 11, especially for P1 constructs. Increasing the pH of the dECM inks resulted in the formation of more dense collagenous networks, consisting of more numerous and thinner fibers. An explanation for this may be that increasing the pH led to the decrease of the net positive charge on each collagen monomer through deprotonating amino acid side chains, which could enhance electrostatic interactions between triple helices and the formation of more dense collagen networks [58,59]. Apart from the pH, the decellularization method was found to influence the mechanical properties of the dECM constructs, with the P1 pH 11 constructs being significantly stiffer compared to the P2 pH 11 constructs, possibly due to the different compositions of the dECM materials derived from the two decellularization protocols. More importantly, the compressive modulus of P1 pH 11 exceeded 150 ​kPa, which is comparable to that found in native meniscus [60,61]. The P1 pH 11 constructs also displayed superior tension-compression nonlinearity compared to P2 pH 11 constructs, which is a biomechanical property critical to load-bearing and transmission in soft tissues like the meniscus [62]. However, the tensile properties of the printed grafts did not reach that of native meniscus tissue, which can be linked to factors such as the overall collagen content of the constructs, collagen cross-linking and the random microstructure of the collagen network, where it is noted that a more defined architecture with radially and circumferentially aligned collagen fibers is found in the native tissue [63].

The evaluation of the biocompatibility of the constructs showed that the dECM constructs supported the attachment and proliferation of fibrochondrocytes, which indicates that the use of chemical crosslinking had a limited effect on the biocompatibility of the dECM biomaterials. Moreover, no significant difference was found in terms of cell viability and proliferation on the constructs prepared at different pH levels, which may be helped by soaking in PBS overnight before cell work. *In vitro* we observed limited cellular infiltration into the body of the dECM constructs, which could be attributed to the dense microstructure. Previous studies, however, have demonstrated cellular remodelling of decellularized meniscus after *in vivo* implantation [64,65], suggesting that 3D printed dECM grafts might be similarly repopulated following implantation into the body. Further *in vivo* studies are required to access the biocompatibility and immune response to these printed grafts, in particular implants printed at high pH, and also to assess the dynamics of recellularization of the dECM implants and how they further mature in orthotopic locations.

Besides providing a bioactive scaffold for meniscus regeneration, reparative scaffolds need to aid in the restoration of the complex organization of the meniscus ECM. Previous studies have shown that the meniscus ECM microarchitecture is essential for mechanical function [66]. The high shear stress during printing has been reported to induce an alignment of the macromolecules, promoting the alignment of the fibers in the final fibrillated hydrogel [67]. Here we found that extrusion of the inks at pH 11 promoted the alignment of the collagen fibrils along the direction of printing, however when patterning pH 7.4 inks this phenomena was not observed. It could be hypothesized that the high pH increased the interaction among the collagen molecules and promoted fibrillogenesis into small collagen fibrils [58], which could be better aligned by the high shear stress during the extrusion process. Further, it was likely that the aligned collagen fibrils guided larger collagen fiber alignment at a physiological temperature, as observed from the polarized light microscopy images. To the best of our knowledge, this study is the first to investigate the use of high concentration dECM inks to generate mechanically functional implants and also to demonstrate the possibility to align the collagen architecture within such constructs using extrusion printing, which could potentially be used to recapitulate the complex architecture of soft tissues such as the meniscus. Future works are, however, required to access the potential of this strategy to print scaled-up implants with complex ECM structures. It can be also speculated that the anisotropic organization of the collagen fibers may improve the tensile properties of the constructs, which warrants further investigation in future studies.

In conclusion, we have generated high concentration dECM inks that display shear thinning and thixotropic properties that makes them ideally suited to 3D printing applications. With additional crosslinking of the dECM inks following thermal gelation at pH 11, it was possible to fabricate highly elastic meniscal tissue equivalents with compressive mechanical properties similar to the native tissue. We believe that this approach could be applied to a wide variety of different biological tissues, enabling the 3D printing of tissue mimics with diverse applications from tissue engineering to surgical planning.

## Credit author statement

**Bin Wang**: Conceptualization, Methodology, Writing – original draft. Visualization, Investigation, Formal analysis; **Xavier Barceló**: Methodology, Investigation, Visualization, Writing – original draft, **Stanislas Von Euw**: Methodology, Investigation, Writing – review & editing, **Daniel J. Kelly**: Conceptualization, Writing – review & editing, Methodology, Supervision, Project administration, Funding acquisition.

## Declaration of competing interest

The authors declare that they have no known competing financial interests or personal relationships that could have appeared to influence the work reported in this paper.

## Data Availability

Data will be made available on request.
